# New Particle Filter Based on GA for Equipment Remaining Useful Life Prediction

**DOI:** 10.3390/s17040696

**Published:** 2017-03-28

**Authors:** Ke Li, Jingjing Wu, Qiuju Zhang, Lei Su, Peng Chen

**Affiliations:** 1 Jiangsu Key Laboratory of Advanced Food Manufacturing Equipment and Technology, Jiangnan University, 1800 Li Hu Avenue, Wuxi 214122, China; dayanlv@live.cn (K.L.); qjzhang@jiangnan.edu.cn (Q.Z.); lei_su2015@jiangnan.edu.cn (L.S.); 2 Graduate School of Bioresources, Mie University/1577 Kurimamachiya-cho, Tsu, Mie 514-8507, Japan; chen@bio.mie-u.ac.jp

**Keywords:** remaining useful life, particle filter, genetic algorithm, starting prediction time, time-varying auto regressive

## Abstract

Remaining useful life (RUL) prediction of equipment has important significance for guaranteeing production efficiency, reducing maintenance cost, and improving plant safety. This paper proposes a novel method based on an new particle filter (PF) for predicting equipment RUL. Genetic algorithm (GA) is employed to improve the particle leanness problem that arises in traditional PF algorithms, and a time-varying auto regressive (TVAR) model and Akaike Information Criterion (AIC) are integrated to establish the dynamic model for PF. Moreover, starting prediction time (SPT) detection method based on hypothesis testing theory is presented, by which SPT of equipment RUL can be adaptively detected. In order to verify the effectiveness of the methods proposed in this study, a simulation test and the accelerating fatigue test of a rolling element bearing are designed for RUL prediction. The test results show the methods proposed in this study can accurately predict the RUL of the rolling element bearing, and it performs better than the traditional PF algorithm and support vector machine (SVM) in the RUL prediction.

## 1. Introduction

Remaining useful life (RUL) prediction of the equipment is the key technology for realizing condition based maintenance (CBM). Equipment RUL plays an important role in the optimization of maintenance decisions. If the equipment RUL is predicted in advance, industrial accidents can be avoided effectively. Thus, RUL prediction of equipment has important significance for guaranteeing production efficiency, reducing maintenance cost, and improving plant safety [1,2,3,4].

In recent years, many works about equipment RUL prediction have been reported. These works can be categorized into model-based and data-driven methods. Model-based methods are to build mathematical models to describe mechanical system and degeneration process of machinery according to physical law. In [5], a bearing RUL was predicted by using Paris crack growth model and GA algorithm. In [6], Oppenheimer et al. constructed a life model based on forman crack growth law of linear elastic fracture mechanics to predict remaining machine life. In [7] a spall progression life model for bearing contact was developed by Choi et al., and it was also combined with the crack initiation life and the crack propagation life models to construct a total life prediction model. However, some parameters in these model-based methods such as initiation crack and crack toughness, need to be obtained by large amounts of history information in advance. Thus, the model-based methods are hardly applied to predict the RUL of machinery in real time.

Data-driven methods derive the degeneration process model of a machine from data measured by sensors online and the model parameters can be adjusted according to real time data. Data-driven methods have strong advantages in RUL prediction, when a mechanical system is complex and mechanical principle is indeterminacy. In [8], Chen et al. proposed a reliability estimation method based on logistic regression model, and applied it to predict the RUL of cutting tool. In [9,10], the artificial neural networks (ANN) were also used in the RUL prediction of cutting tool. In [1], Dong et al. proposed a statistical model methodology based on segmental hidden semi-Markov models (HSMMs) to develop a prognosis for the RUL of the hydraulic pumps. In [2], Qian et al. estimated a bearing degradation by using recurrence quantification analysis and Kalman filter. In addition, some relevant reaches on the data-driven methods, such as neural network, support vector machine, relevance vector machine, and Bayesian algorithm can be seen in [11,12,13,14,15].

Among all the data-driven methods, the particle filter (PF) technique is one of the most effective for nonlinear and non-Gaussian systems, which can accurately estimate the future state of mechanical systems. In the last decade, PF technique has been widely applied in diagnosis and prognosis fields. In [16], Orchard and Vachtsevanos utilized PF algorithm to estimate the state probability density function of a planetary gear in real-time. In [17], Orchard et al. applied a PF algorithm to predict state of charge of lithium-ion batteries. In [18], Abbas et al. used a PF algorithm in failure prognosis of the electrical components in automotive systems. In [19], bearing fault signal noise reduction processing was carried out with a PF algorithm and AR model. In [20], the Rao-Blackwellized PF was combined with the unscented transform for fault diagnosis and detection of a nonlinear system. However, there exists some shortcomings in the traditional PF algorithm. Particle leanness is one of the major drawbacks, and it may lead to misestimation of future states. The simple method to solve the particle leanness problem is to increase the number of particles. However, computing burdens of PF will be increased. If computing time of PF is too long, PF is difficult to apply in practical engineering.

For the above reasons, this paper presents a novel equipment RUL prediction method based on the PF algorithm and genetic algorithm (GA). The major contributions of this paper are: (1) The reason of particle leanness arises in traditional PF algorithm is analyzed, and the new PF called GA-PF is presented to solve the particle leanness problem. Compare the GA-PF with the traditional PF, it can provide more accurate RUL prediction results; (2) Starting prediction time (SPT) detection method based on hypothesis testing theory is presented, by which SPT of equipment RUL can be adaptively detected.

The remaining part of this paper is organized as follows; Section 2 introduces the theory of the traditional PF algorithm, and the GA-PF is also described in detail; Section 3 shows the RUL prediction framework that is constructed by using the methods proposed in this paper; In Section 4, a simulation test is designed for evaluating the performance of the RUL prediction method; In Section 5, the accelerating fatigue test of a rolling element bearing verifies the effectiveness of the methods proposed in this study; Finally, conclusions are summarized in Section 6.

## 2. Particle Filter

### 2.1. Basic Theory of PF

PF is recursive Bayesian filtering estimation based on Monte Carlo algorithm [21]. It can approximate posterior probability density by a set of weighted samples also known as particles.

The state space model of a nonlinear system is represented as follows:

(1) $$x_{t}=f_{t}(x_{t-1},v_{t})$$

(2) $$z_{t}=h_{t}(x_{t},w_{t})$$

where, Equations (1) and (2) are called state equation and observation equation, respectively; *x_t_* and *z_t_* express state and observation vector of the system at *t* time, respectively; *v_t_* and *w_t_* are state noise and observation noise, respectively; *f_t_* and *h_t_* are nonlinear functions.

Assuming the state vector *x_t_* follows one-order Markov process, the prior probability function of *x_t_* can be derived by Chapman-Kolmogorov equation,

(3) $$\begin{array}{c} p(x_{t}|z_{0:t-1})=\int p(x_{t}|x_{t-1},z_{0:t-1})p(x_{t-1}|z_{0:t-1})dx_{t-1}\\ =\int p(x_{t}|x_{t-1})p(x_{t-1}|z_{0:t-1})dx_{t-1} \end{array}$$

where, the notation “:” in *Z*_0:t−1_ expresses that from 0 to t − 1.

Updating posterior probability of *x_t_* by *z_t_*

(4) $$p(x_{t}|z_{0:t})=\frac{p(x_{t}|z_{0:t-1})p(z_{t}|x_{t})}{p(z_{t}|z_{0:t-1})}$$

where,

(5) $$p(z_{t}|z_{0:t-1})=\int p(x_{t}|z_{0:t-1})p(z_{t}|x_{t})dx_{t}$$

Recursive Bayesian filtering consists of Equations (3) and (4). However, in most cases, it is difficult to obtain posterior probability *p(x_t_*|*z*_0*:t*_*)* by the analytic solution. Instead of the analytic solution, PF based Monte Carlo algorithm is introduced.

The key idea of PF is to approximate posterior probability density by a set of weighted particles (samples). Using *N* particles {*x^i^_0:__t_* ,*ω^i^_t_*}*^N^_i = 1_* to express the posterior probability *p(x_0:t_*|*z_0:t_)*, {*x_0:_^i^_t_*, *i =* 0,1…*N*} is the particle set, and sampled from the posterior probability distribution *p(x_0:t − 1_*|*z_0:t − 1_)*. The weight of each particle is {*ω^i^_t_*, *i* = 0,1…*N*}, and ∑*^N^_i = 1_ ω^i^_t_* = 1. Hence, the posterior probability *p(x_0:t_*|*z_0:t_)* at time *t* can be approximated by *N* particles as follows

(6) $$p(x_{0:t}|z_{0:t})\approx \sum_{i=1}^{N}\omega_{t}^{i}\delta (x_{0:t}-x_{0:t}^{i})$$

where *δ*(·) is Dirac-delta function

(7) $$\delta (x_{0:t}-x_{0:t}^{i})=\{\begin{array}{cc} 1 & x_{0:t}=x_{0:t}^{i}\\ 0 & x_{0:t}\neq x_{0:t}^{i} \end{array}$$

Since it is difficult to obtain samples from the posterior probability distribution directly, the importance of a sampling method that can easily obtain samples from an importance distribution is adopted.

The importance distribution is given as

(8) $$q(x_{0:t}|z_{0:t})=q(x_{t}|x_{t-1},z_{t})q(x_{0:t-1}|z_{0:t-1})$$

The importance weight *ω^i^_t_* for each sample is as follows

(9) $$\omega_{t}^{i}=\frac{p(x_{0:t}|z_{0:t})}{q(x_{0:t}|z_{0:t})}\propto \omega_{t-1}^{i}\frac{p(z_{t}|x_{t}^{i})p(x_{t}^{i}|x_{t-1}^{i})}{q(x_{t}^{i}|x_{0:t-1}^{i},z_{1:t})}$$

where, the symbol ∝ indicates to be proportional to.

The normalized importance weight ${\tilde{\omega }}_{t}^{i}$ is

(10) $${\tilde{\omega }}_{t}^{i}=\frac{\omega_{t}^{i}}{\sum_{i=1}^{N}\omega_{t}^{i}}$$

The state *x_t_* can be estimated as

(11) $${\hat{x}}_{t}\approx \sum_{i=1}^{N}{\tilde{\omega }}_{t}^{i}x_{t}^{i}$$

Using the above method, the state *x_t_* can be estimated by the posterior probability. However, with increasing iterations, the variance of particle weight will become great, most of particles have small weight. This results in particle degeneracy of the PF algorithm [22]. To solve the particle degeneracy problem, multinomial resampling technique is utilized, and its procedure is shown as follows.

Multinomial resampling:

Step 1: For *i* = 1,2, … *N*, draw a sample set {*u_i_*} from the uniformly distribution (0,1];

Step 2: For *i* = 1,2, …*N*, find the variable quantity *j* (*j* = 1,2, …*N*) that satisfies

(12) $$\sum_{n=1}^{j-1}\omega_{n}<u_{i}\leq \sum_{n=1}^{j}\omega_{n}$$

Step 3: Store the *x^i^_t_* as a new particle, and the weight of the new particle *ω^i^_t_* = 1/*N*.

The degree of particle degeneracy can be appraised using the effective sample size *N_eff_* [23]

(13) $$N_{eff}=\frac{1}{\sum_{i=1}^{N}{\left({\tilde{\omega }}_{t}^{i}\right)}^{2}}$$

**Figure 1:**
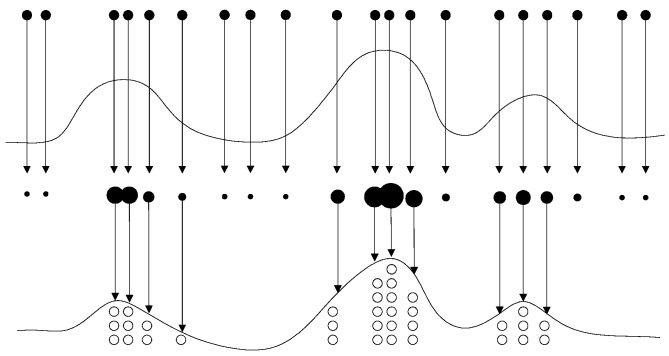
Illustration of resampling.

From Equation (13), the smaller value of the effective sample size *N_eff_*, the more serious the problem of particle degradation will be. Setting the threshold of effective sample *N_r_*, *N_r_* = *m* × *N*, *m* ∈ (0,1). If *N_eff_* < *N_r_* particle degradation is serious, PF system performs resampling. After the resampling process, the particles that have small weight are knocked out, and many new particles are obtained by the particles that have big weight, as shown in Figure 1.

The resampling method solves the problem of particle degradation, many new particles are produced. However, the resampling method brings a new problem. In resampling procedure, only the particles that have big weight are selected as copy samples, the new particles are replicas of them, and the particles with small weight are eliminated. In most cases, all the resampling operations are focused on a few particles with big weight, the particles lose diversity. This problem is also called particle leanness.

### 2.2. PF Based on GA

GA is an artificial intelligent algorithm based on natural selection and genetic mechanism of biological organism evolutionary process [24]. GA mainly includes selection, crossover, and mutation operations. In GA, every individual fitness degree is evaluated by using fitness function. The individuals that have high fitness degree values will be retained, conversely, the individuals that have low fitness degree values will be eliminated. After the selection operation, the retained individuals are used to reproduce offspring as parents on the basis of a crossover rate. Lastly, the mutation operator is carried out by changing some information of offspring individuals. After the above operations, the new offspring are obtained and the diversity of population is improved. In addition, the features of the good individuals are passed on to the new offspring.

In this study, the GA method is introduced to replace the traditional resampling operator for easing the particle leanness problem. The procedure of GA resampling is explained as follows:

Selection operator: In PF, the smaller variance of the particle weight, the state estimation more accurate will be, thus a selection method is designed according to the variance of the particle weight. Assume ${\left\{x_{k}^{i},{\tilde{\omega }}_{k}^{i}\right\}}_{i=1}^{N}$ is a particle set obtained at time *k*, and ${\tilde{\omega }}_{k}^{i}$ indicates particle weight. *V_k_* indicates the variance of the particle weight. *q* is a threshold which set in advance. If *V_k_* > *q*, the crossover and mutation operators need to be performed.

Crossover operation: Separate the particles into two sets by the strategy explained as follows.

(14) $$A=round(\frac{1}{\sum_{i=1}^{N}{\left({\tilde{\omega }}_{k}^{i}\right)}^{2}})$$

where, round(⋅) indicates the operation which round to the nearest integer. All particles are sorted with the weight values in descending order. The top *A* particles with large weight are divided into set *B,* and the other particles are divided into set *S*. Two particles *x^m^_k_*, *x^n^_k_* are randomly selected form sets *B* and *S* respectively, and the crossover operator is carried out by using Equations (15) and (16).

(15) $${\tilde{x}}_{k}^{n}=\beta x_{k}^{n}+(1-\beta )x_{k}^{m}$$

where, *β* is parameter which determine the amount of information being exchanged, and can be calculated as follows

(16) $$\beta =\frac{{\tilde{\omega }}_{i}^{n}}{{\tilde{\omega }}_{i}^{m}+{\tilde{\omega }}_{i}^{n}}$$

Mutation operation: In order to better increase particle diversity, a mutation operator is designed. The mutation operation is performed on the new particles produced in crossover operation.

(17) $${\hat{x}}_{k}^{n}=\{\begin{array}{cc} 2x_{k}^{m}-{\tilde{x}}_{k}^{n} & P\geq R\\ {\tilde{x}}_{k}^{n} & P<R \end{array}$$

where, *R* ∈ [0,1] is a random variable; *P* indicates the mutation probability.

After the GA resampling step, the particles in the low probability density region are adjusted to the high probability density region, and the particle diversity is also improved. The whole procedure of the GA-PF is presented as follows:

Step 1: Setting time *k* = 0, for *i* = 1,2,…*N*, draw the particles *x^i^_0_* from the prior probability distribution, and all of the particles weight are 1/*N*;

Step 2: At time *k* > 0, for *i* = 1,2,3,…*N*, draw the particles *x^i^_k_* from the importance distribution, and calculate the particle weight *ω^i^_k_* by Equation (9), and normalize the particle weight *ω^i^_k_* by Equation (10);

Step 3: Perform the selection, crossover, and mutation operations, and normalize the particle weight again;

Step 4: For *i* = 1,2,…*N*, perform the state estimation with Equation (11);

Step 5: Set *k* = *k* + 1, and obtain new observation data, return to step (2).

## 3. RUL Prediction Algorithm

**Figure 2:**
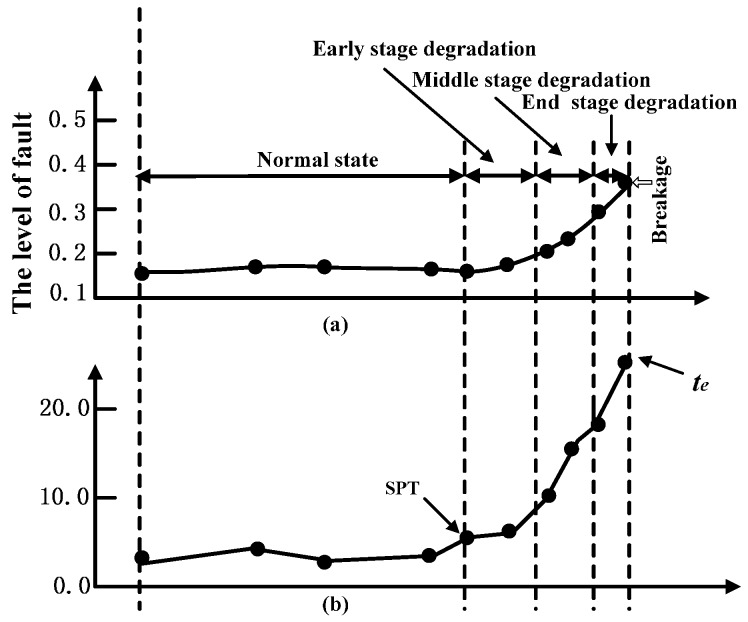
Five stages of the whole machine life.

In general, the whole machine life can be divided into five stages as shown in Figure 2: (1) normal stage; (2) early degradation stage; (3) middle degradation stage; (4) terminal degradation stage; (5) breakdown stage [25]. The life prediction of machine is often stared from early degradation stage. However, when the machine is in the early abnormal stage, or the machine fault is slight, the change of the vibration data is small, it is difficult to detect the starting time of the occurring degradation stage by watching the graph of observation data. To deal with the above shortcoming, this study proposes an SPT detection method based hypothesis testing theory.

Assuming that *V_i,m_* is vibration data set collected from time *t*_1_ to *t_n_*. *i* indicates data acquisition time, and *i* = 1,2…*n*; *m* represents the number of data acquisition. Then the vibration data measured from time *t*_1_ to *t_n−_*_1_ and the vibration data measured at time *t_n_* can be expressed as *V_(_*_1*~n*−1*),m*_ and *V_n,m_*, respectively. The significant difference between *V_(_*_1*~n−*1*),m*_ and *V_n,m_* can be detected by using Equation (18).

(18) $$R=({\bar{V}}_{n,m}-{\bar{V}}_{(n-1),m})\sqrt{\frac{N_{1}N_{2}\varphi }{\left(N_{1}+N_{2})(N_{1}S_{1}^{2}+N_{2}S_{2}^{2}\right)}}$$

where, ${\bar{V}}_{n,m}$ and ${\bar{V}}_{(1\sim n-1),m}$ are the average values of *V_n,m_* and *V*_(1*~n−*1*),m*_ respectively, and ${\bar{V}}_{n,m}=\sum_{1}^{m}V_{n,m}/m$, ${\bar{V}}_{(1\sim n-1),m}=\sum_{i=1}^{n-1}\sum_{1}^{m}V_{im}/m(n-1)$; *S*_1_ and *S*_2_ express standard deviation of *V_(_*_1*~n−*1*),m*_ and *V_n,m_* respectively; *N*_1_ and *N*_2_ indicate the size of *V_(_*_1*~n−*1*),m*_ and *V_n,m_*, respectively.

If *R* satisfies Equation (19), there is a significant difference between *V_(_*_1*~n−*1*),m*_ and *V_n,m_*. That is to say, *t_n_* is judged as machine occurring degradation time, and machine tendency prediction and useful life prediction are stated from the point of time *t_n_*.

(19) $$R>T(N_{1}+N_{2}-2,a/2)$$

where, α represents the level of significance, and α = 0.05.

To use the PF technique for machine tendency prediction and RUL prediction, the state model and observation model described in Equations (1) and (2) need to be established first. In this study, TVAR model is chosen to construct the time series model of mechanical vibration signal. Assuming *x_t_* is vibration signal, the TVAR model of vibration signal is given as follows.

(20) $$x_{t}=-\sum_{i=1}^{p}a_{i}(t)x_{t-i}+e_{t}$$

where, *a_i_* indicates time-varying coefficient; *p* denotes model order; *e_t_* is residual error of the model.

*a_i_* is a linear combination of a set of basic function,

(21) $$a_{j}(t)=-\sum_{j=0}^{m}a_{ij}g_{j}(t)$$

where, *m* is extended dimension; *a_ij_* is weight of basis function; *g_j_(t)* express time basis function. This study chooses DCT basis function shown in Equation (22) to estimate the time-varying coefficient of the TVAR model.

(22) $$g_{j}(t)=a(t)\cos(j\pi (2k+1)/2N)$$

(23) $$a(t)=\{\begin{array}{cc} \sqrt{1/N} & t=0\\ \sqrt{2/N} & t\neq 0 \end{array}$$

In this paper, Akaike Information Criterion (AIC) method is introduced for determining model order *p*, and mathematical expression of AIC is given as follows. The model with the smallest AIC value is the most suitable model for the time series. A detailed description about AIC method can be found in reference [26].

(24) $$AIC(p)=\ln{\hat{\sigma }}^{2}+\frac{2p}{N-r}$$

where, *N* is the length of signal; *r* indicates pre-order of the model.

In addition, the observation model is determined as

(25) $$Z_{t}=x_{t}+w_{t}$$

where, *x_t_* and *z_t_* express state and observation values at time *t*; *w_t_* is observation noise.

After SPT is decided and TVAR model is constructed, the GA-PF introduced in Section 2 is used to predict machine state and RUL. RUL of machine is calculated as

(26) $$RUI_{P}=t_{e}-t_{p}$$

where, *t_p_* is present prediction time; *t_e_* indicates the end of lifetime of machine.

**Figure 3:**
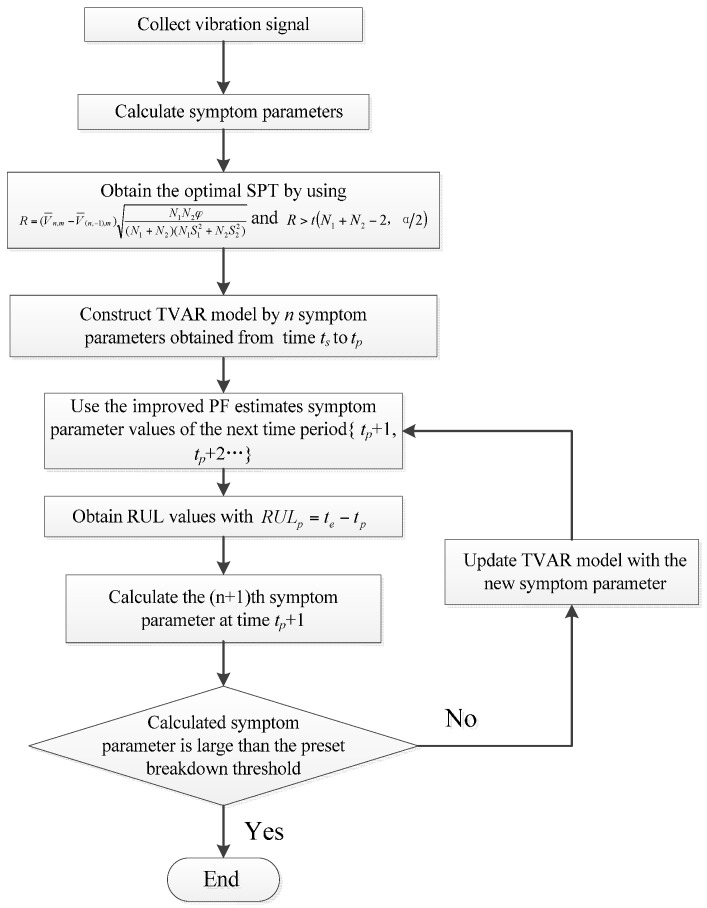
Flowchart of the equipment RUL prediction.

The flowchart of RUL prediction by the GA-PF algorithm is shown in Figure 3 and the procedure is explained as follows:

Step 1: The vibration signal is collected, and the symptom parameters that can sensitively reflect machine state are calculated;

Step 2: By using Equations (18) and (19), the optimal SPT is obtained;

Step 3: Assuming the optimal SPT and the present prediction time are *t_s_* and *t_p_* respectively; TVAR model is constructed by *n* symptom parameters obtained from time *t_s_* to *t_p_*;

Step 4: Use the GA-PF to estimate symptom parameter values of the next time period {*t_p_* + 1, *t_p_* + 2…} until the estimated symptom parameter is larger than the preset breakdown threshold;

Step 5: RUL value is obtained with Equation (26);

Step 6: Calculating the (*n* + 1)th symptom parameter at time *t_p_* + 1, and TVAR model is updated with the new symptom parameter;

Step 7: The new estimation is conducted by using the GA-PF and the updated TVAR model until the estimated symptom parameter is larger than the preset breakdown threshold, and the new RUL value is obtained with Equation (26);

Step 8: Loop Step 3 to Step 7 until the calculated symptom parameter value reaches the breakdown threshold.

## 4. Simulation

**Figure 4:**
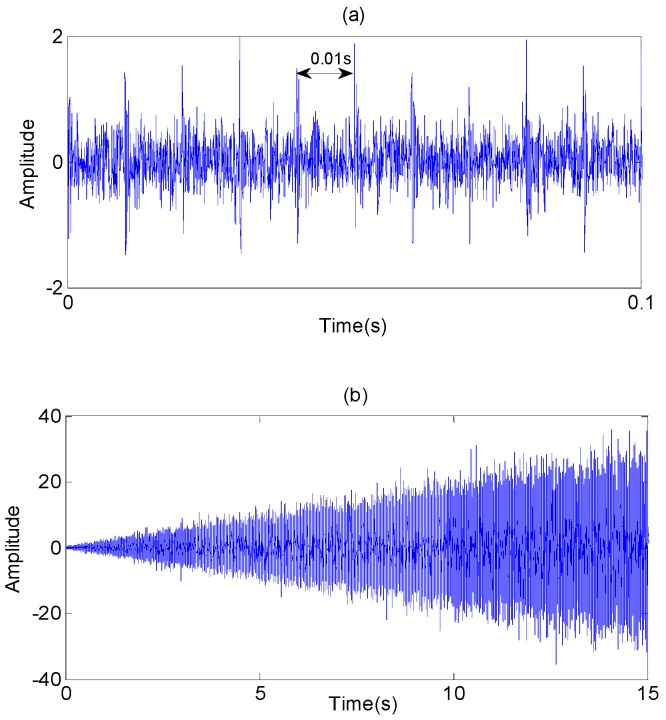
Simulation vibration data. (**a**) the vibration data in early degradation stage; (**b**) the vibration data of the whole degradation stage.

**Figure 5:**
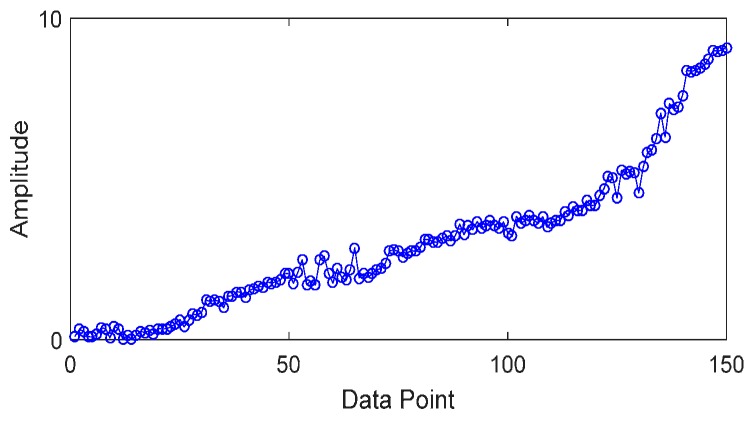
RMS of simulation vibration data.

In order to evaluate the effectiveness of the RUL prediction method, a simulation test is designed. We simulate vibration data of accelerating fatigue of a rolling element bearing by using MATLAB. The vibration signal is expressed as Equation (27), and parameters of the vibration signal are as follows: natural frequency *ω_n_* = 3 kHz; sampling frequency *f_s_* = 20 kHz; damped coefficient *ε* = 0.1; displacement constant *x* = 2; impact interval is 0.01s; sampling points are 2048. In order to simulate the whole degradation process of the bearing, the signals are repeated 150 times with the increasing fault severity, and Figure 4 shows the vibration signals of the whole lifetime. As shown in Figure 5, root mean square (RMS) is calculated from the simulation vibration data to reflect the degradation process of rolling element bearing.

(27) $$y(t)=xe^{-\varepsilon \omega_{n}}\sin\omega_{n}\sqrt{1-\varepsilon^{2}}t+v(t)$$

where, *v(t)* indicates white noise.

**Figure 6:**
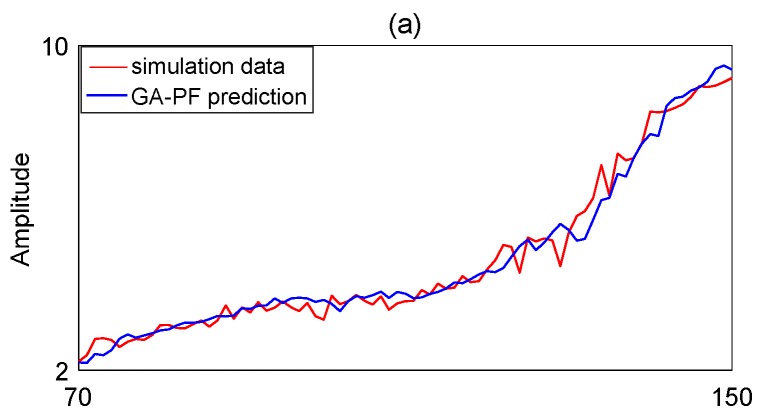

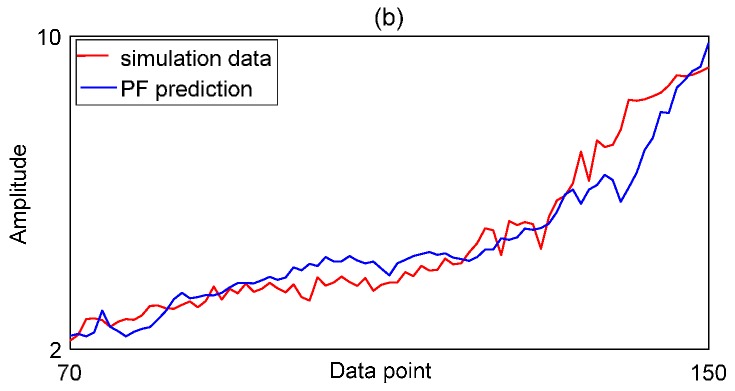
Prediction results. (**a**) GA-PF; (**b**) general PF.

In this simulation test, the first 70 RMS data are utilized to construct TVAR model, and the remaining 80 data points are predicted by the general PF and the GA-PF, respectively. For GA-PF in the simulation, the threshold *q* = 0.05; the mutation probability *P* = 0.5; the number of particle is set to 1000; Figure 6a,b shows the prediction results by the general PF and the GA-PF, respectively. Root mean square error (RMSE), as shown in Equation (28), and the degree of particle degeneracy are used to evaluate the performance of the general PF and the GA-PF. The degree of particle degeneracy can be appraised using the effective sample size *N_eff_* as shown in Equation (13). The comparison results are listed in Table 1. To see Table 1, with the increase of particle, the accuracy of prediction is improved, and the running time is also increased. Considering the time and accuracy, we selected particle number *N* = 1000. When *N* = 1000, RMSE of the GA-PF and the general PF are 0.1865 and 0.3126, the running times are 1.232 s and 1.139 s, respectively. Although running time of GA-PF is 0.093 s longer than general PF, but the prediction accuracy of GA-PF is much higher than general PF. Moreover, available particles of the GA-PF and the general PF are 413 and 205, respectively. This means that the GA-PF can effectively prevent particle degeneracy and increase particle diversity. Thus, the performance of GA-PF is better than general PF, the former is more suitable for equipment RUL prediction.

(28) $$RMSE=\sqrt{\frac{1}{n}\sum_{i=1}^{n}{\left(x_{i}-{\hat{x}}_{i}\right)}^{2}}$$

where, *x_i_* is the simulation data. ${\hat{x}}_{i}$ is estimate data. *n* is length of the data.

## 5. Application to RUL of Rolling Element Bearing

**Figure 7:**
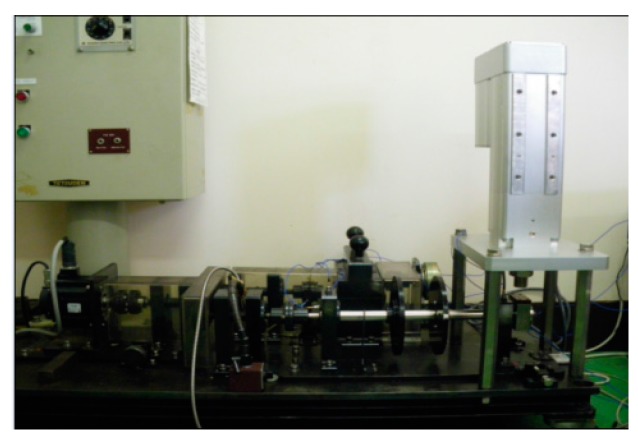
Illustration of the experimental setup for test.

**Figure 8:**
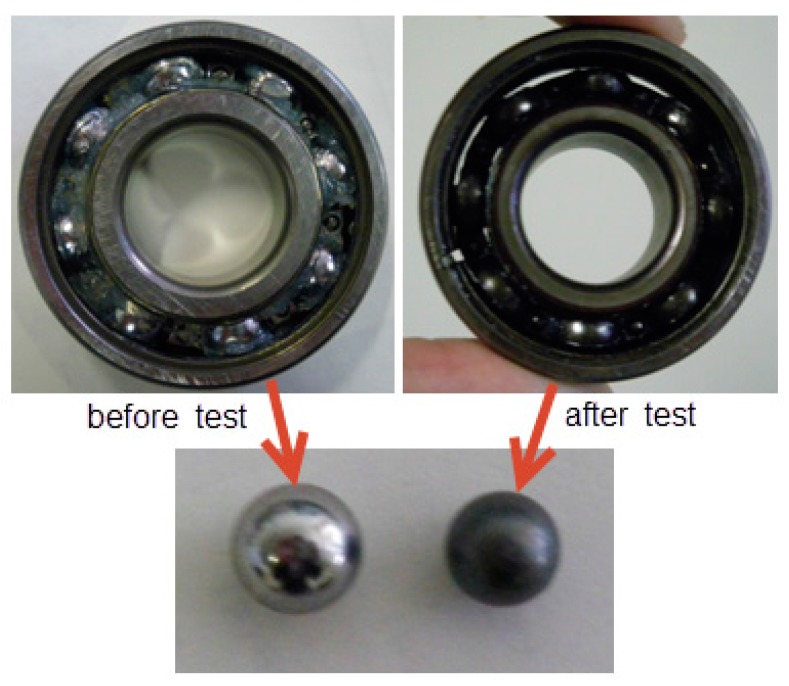
Bearing before and after test.

**Figure 9:**
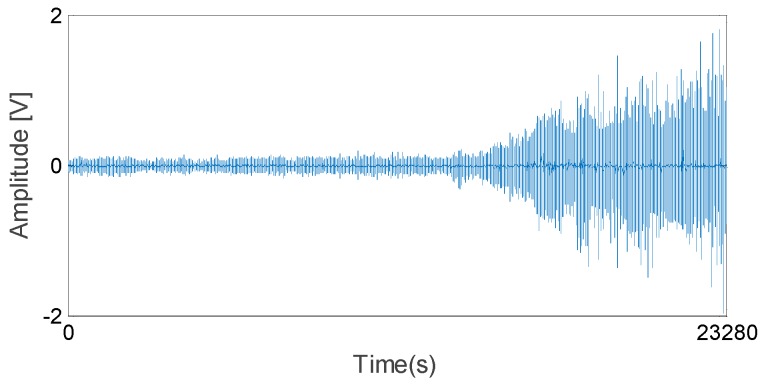
Vibration signal of the whole useful time.

In this section, a rolling element bearing RUL prediction is designed to verify the effectiveness of the methods proposed in this study. The experimental system for bearing RUL prediction is constructed as shown in Figure 7. The bearing used for RUL prediction test is N205 with eight rollers, pitch diameter: 42 mm, roller diameter: 7.5 mm, and tapered contact angle: 0°. Maximum dynamic load of N205 is 17.7 kN, to carry out accelerating fatigue tests of the bearing in a few hours, 17 kN load is exerted on the test bearing by the loading equipment (Shizuoka RCS2-RA13R, IAI Co. Ltd., Shizuoka, Japan). Figure 8 shows the appearance of the bearing before and after test. During accelerating fatigue test, the rotation speed of the test system is set at a constant speed of 800 rpm. Accelerometers (PCB ICP Model 480C02, PCB Piezotronics Inc., New York, NY, USA) are fixed on the bearing housing to measure the raw vibration signals. The sampling frequency is 20 kHz. The vibration data is measured every 2 min, each sample includes 20,480 data points. In total, 258 data files are obtained. After being amplified by the signal conditioner, the vibration signals are transmitted into the computer for storage and data handing by the data acquisition (KEYENCE NR-200, YOKOGAWA Company Limited, Tokyo, Japan). When the vibration signal amplitude is greater than 2, the useful life of the bearing ends. Figure 9 shows the vibration signal of the whole useful time. As shown in Figure 9, the vibration signal amplitude exceeds 2 at time 23,280 s, in other words, the whole life of the test bearing is 23,280 s.

**Figure 10:**
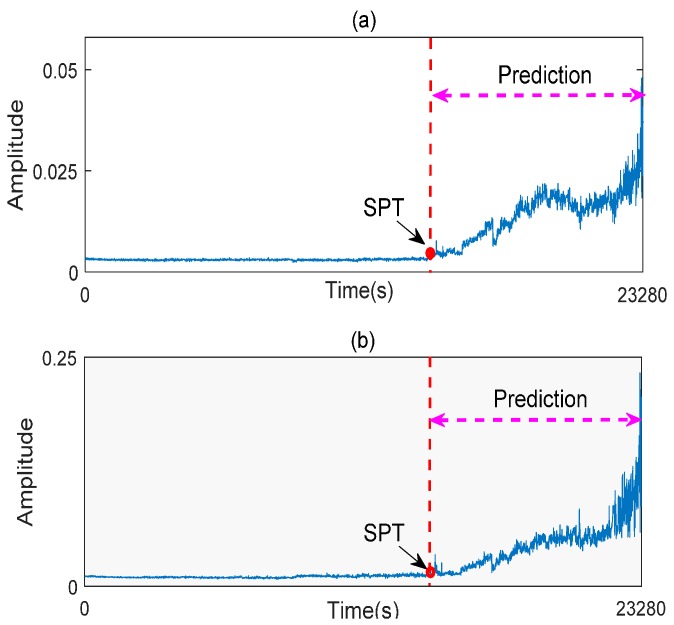
Bearing degradation curves (**a**) RMS; and (**b**) peak value.

In this study, root mean square (RMS) and peak values calculated from the raw vibration signal are chosen to reflect degradation process of the bearing. The reason is that RMS and peak values can sensitively reflect the increase of vibration signal energy with the development of the bearing fault. Figure 10 shows the bearing degradation curves composed by RMS and peak values.

**Figure 11:**
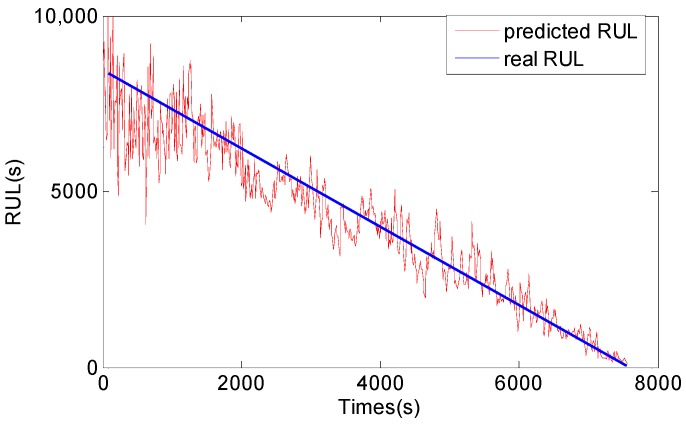
RMS RUL prediction result by the GA-PF.

**Figure 12:**
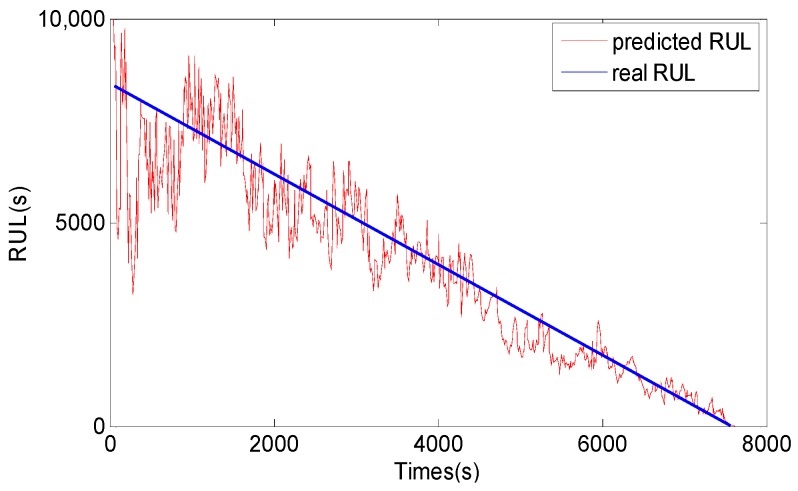
Peak value RUL prediction result by the GA-PF.

After vibration signals collection and symptom parameters extraction, RUL prediction of the rolling element bearing is carried out by using the methods proposed in this study. Firstly, SPT for bearing RUL prediction is detected by the SPT detection approach introduced in Section 3. As shown in Figure 10a, the SPT of RMS is detected at 15,840 s. Secondly, TVAR model is constructed with the first 600 s RMS data from 15,130 s to 15,730 s, and the model order is decided with the smallest AIC value. Lastly, the GA-PF algorithm with 1000 particles is used to predict RUL of the bearing. The result of the bearing RUL prediction with RMS data is shown in Figure 11. Similar to RMS, peak values shown in Figure 10b are also calculated to predict the bearing RUL, and the SPT of peak values are detected at 15,080 s. TVAR model of peak value is constructed with the data from 15,080 s to 15,680 s. The peak values prediction results are shown in Figure 12. In the Figure 11 and Figure 12, *x*-coordinate indicates prediction times from SPT to the end of the bearing life, and y-coordinate represents the predicted RUL. To see Figure 11 and Figure 12, the RUL prediction of RMS and peak values can accurately reflect the changing trend of the bearing RUL. At the beginning of the RUL prediction, both the RMS and peak values form relatively large prediction errors because of a lack of prediction data. With the increase of the RMS and peak value data used in the prediction model, the prediction error fluctuation is gradually reduced, and the predicted RUL is close to the real bearing RUL values.

**Figure 13:**
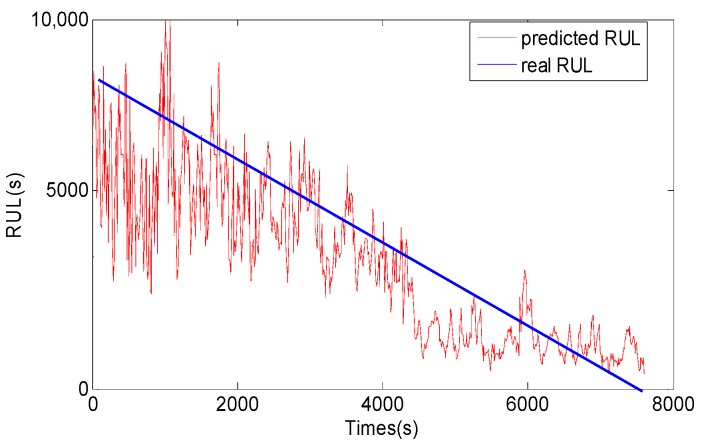
RMS RUL prediction result by the general PF.

**Figure 14:**
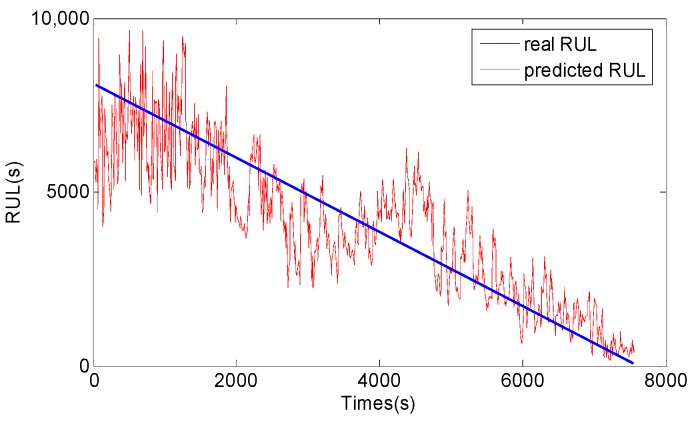
Peak value RUL prediction result by the general PF.

**Figure 15:**
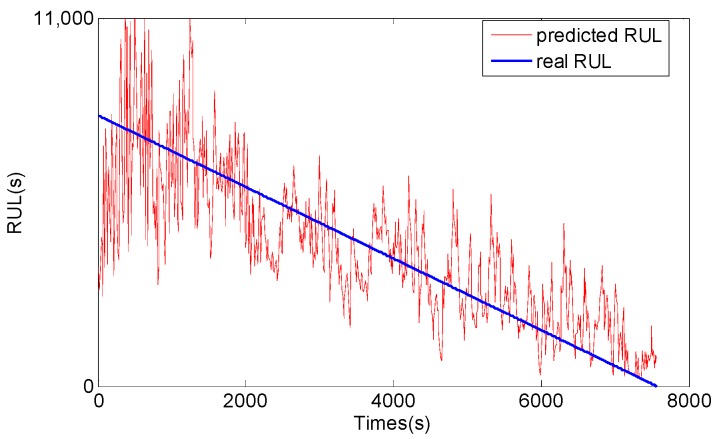
RMS RUL prediction result by SVM.

**Figure 16:**
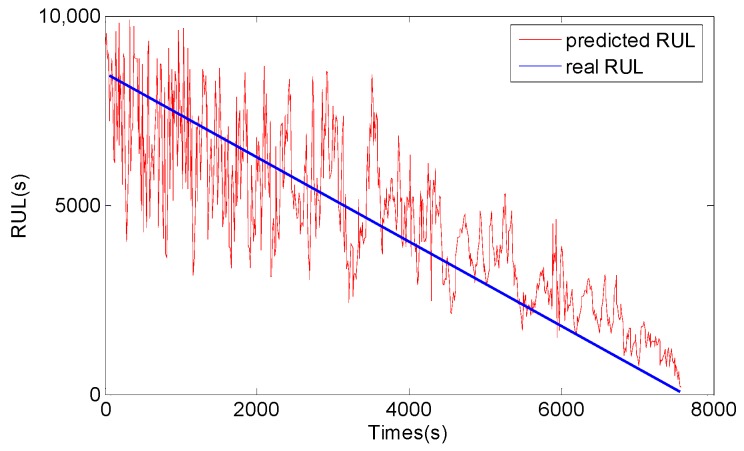
Peak value RUL prediction result by SVM.

For comparison, the general PF and support vector machine (SVM) methods are also used to predict RUL of the bearing, and the results are shown in Figure 13, Figure 14, Figure 15 and Figure 16. The performance of three methods is evaluated based on RMSE, mean relative error (MRE), variance relative error (VRE), and the effective particle number, and comparison results are listed in Table 2. To see Figure 11, Figure 12, Figure 13, Figure 14, Figure 15 and Figure 16 and Table 2, although the general PF and SVM methods can also reflect the changing trend of the bearing RUL, the prediction errors of the bearing RUL obtained by both the general PF and SVM methods are larger than the GA-PF. The reasons can be explained as (1) the general PF has a particle leanness problem, and cannot estimate the state accurately; (2) The kernel function and regression parameters of SVM may not be the optimal choice and do not meet the requirements of RUL prediction. Moreover, the available particle number of the GA-PF is larger than that of the general PF, which means that the GA-PF can effectively solve the problem of particle degradation and keep the particle diversity. From the experimental results, we can see that the proposed methods in this paper are effective, and can be used for equipment RUL prediction.

(29) $$MRE=\frac{1}{n}\sum_{i=1}^{n}\left|\frac{x_{i}-{\hat{x}}_{i}}{x_{i}}\right|$$

(30) $$VRE=\frac{1}{n}\sum_{i=1}^{n}(\left|\frac{x_{i}-{\hat{x}}_{i}}{x_{i}}\right|-E_{MRE}{)}^{2}$$

## 6. Conclusions

In order to accurately predict the equipment RUL, this paper proposed a new RUL prediction method based on the GA-PF algorithm. GA was employed to replace the traditional resampling operator for solving the particle leanness problem. By using crossover and mutation operators of GA, the particles in the low probability density region were adjusted to the high probability density region, and the particle diversity was improved. Moreover, SPT of equipment RUL was adaptively detected by a method based on hypothesis testing theory. Both the simulation test and the accelerating fatigue test of a rolling element bearing were designed to verify the effectiveness of the methods proposed in this study. The test results showed that the methods proposed in this study could accurately predict the RUL of the rolling element bearing, and it performed better than the traditional PF algorithm in the RUL prediction.

## Figures and Tables

**Table 1:** Comparison results.

	Particle Number	RMSE	Available Particle	Time
General PF	500	0.7852	127	0.456
	1000	0.3126	205	1.139
	2000	0.2816	276	3.013
	500	0.3092	215	0.541
GA-PF	1000	0.1865	413	1.232
	2000	0.1772	501	3.251

**Table 2:** Comparison of results of different methods.

SP	Method	RMSE	MRE	VRE	Available Particle
RMS	SVM	4.78	0.63	4.75	
RMS	General PF	4.57	0.58	1.75	233
RMS	GA-PF	2.21	0.18	0.13	452
Peak value	SVM	7.15	1.07	9.86	
Peak value	General PF	6.58	0.92	3.11	216
Peak value	GA-PF	3.19	0.25	0.05	437

## References

[1] Malhi A., Yan R., Gao R.X. (2011). Prognosis of defect propagation based on recurrent neural networks. IEEE Trans. Instrum. Meas..

[2] Qian Y., Yan R., Hu S. (2014). Bearing degradation evaluation using recurrence quantification analysis and Kalman filter. IEEE Trans. Instrum. Meas..

[3] Li K., Chen P., Wang S.M. (2012). An Intelligent Diagnosis Method for Rotating Machinery Using Least Squares Mapping and a Fuzzy Neural Network. Sensors.

[4] Liao L.X. (2014). Discovering prognostic features using genetic programmingin remaining useful life prediction. IEEE Trans. Ind. Electron..

[5] Oppenheimer C.H., Loparo K.A. (2002). Physically based diagnosis and prognosis of cracked rotor shafts. Proc. SPIE.

[6] Choi Y., Liu C.R. (2007). Spall progression life model for rolling contact verified by finish hard machined surfaces. Wear.

[7] Chen B., Chen X., Li B., Cai G. (2011). Reliability estimation for cutting tool based on Logistic Regression Model. Chin. J. Mech. Eng..

[8] Nakai M.E., Aguiar P.R., Junior H.G., Bianchi E.C., Spatti D., D’Addona D.M. (2015). Evaluation of Neural Models Applied to the Estimation of Tool Wear in the Grinding of Advanced Ceramics. Expert Syst. Appl..

[9] D’Addona D.M., Matarazzo D., Ullah A.M.M.S., Teti R. (2015). Tool wear control through cognitive paradigms. Procedia CIRP.

[10] Dong M., He D. (2007). A segmental hidden semi-Markov model (HSMM)-based diagnostics and prognostics framework and methodology. Mech. Syst. Signal Process..

[11] Chen C.C., Zhang B., Vachtsevanos G., Orchard M.E. (2011). Machine condition prediction based on adaptive neuro-fuzzy and high-order particle filtering. IEEE Trans. Ind. Electron..

[12] Maio F.D., Tsui K.L., Zio E. (2012). Combining relevance vector machines and exponential regression for bearing residual life estimation. Mech. Syst. Signal Process..

[13] Chen X., Shen Z., He Z., Sun C., Liu Z. (2013). Remaining life prognostics of rolling bearing based on relative features and multivariable support vector machine. Proc. Inst. Mech. Eng. C J. Mech. Eng. Sci..

[14] Chen C., Zhang B., Vachtsevanos G. (2012). Prediction of machine health condition using Neuro-fuzzy and Bayesian algorithms. IEEE Trans. Instrum. Meas..

[15] Orchard M.E., Vachtsevanos G.J. (2009). A particle-filtering approach foron-line fault diagnosis and failure prognosis. Trans. Inst. Meas. Control.

[16] Orchard M.E., Hevia-Koch P., Zhang B., Tang L. (2013). Risk measures for particle-filtering-based state-of-charge prognosis in lithium-ion batteries. IEEE Trans. Ind. Electron..

[17] Abbas M., Ferri A., Orchard M.E., Vachtsevanos G. An intelligent diagnostic/prognostic framework for automotive electrical systems. Proceedings of the IEEE Intelligent Vehicle Symposium.

[18] Zhang H.B. (2016). Dynamic Auto-regression Prediction Model Based on Particle Filter. Electr. Sci. Technol..

[19] Liu Y., Sun D.Q., Kong L. Rao-blackwellized particle filtering for fault detection and diagnosis. Proceedings of the 29th Chinese Control Conference.

[20] Doucet A., de Freitas N., Neil G. (2001). Sequential Monte-Carlo Methods in Practice.

[21] Douc R., Cappé O. Comparison of resampling schemes for particle filtering. Proceedings of the 4th Intelligence Symposium on Image Signal Processing and Analysis.

[22] Arulampalam M.S., Maskell S., Gordon N., Clapp T. (2002). A tutorial on particle filters for online nonlinear/non-Gaussian Bayesian tracking. IEEE Trans. Signal Process..

[23] Yoon Y., Kim Y. (2013). An efficient genetic algorithm for maximum coverage deployment in wireless sensor networks. IEEE Trans. Cybern..

[24] Chen P., Koide Y., Li K., Satonaga N. (2011). Life Prediction of Rolling Bearing Using Genetic Algorithm. Appl. Mech. Mater..

[25] Fei W.C., Bai L. (2009). Time-varying parameter auto-regressive models for autocovariance nonstationary time series. Sci. China Ser. A Math..

[26] Liu Y., He B., Liu F., Lu S., Zhao Y. (2016). Feature fusion using kernel joint approximate diagonalization of eigen-matrices for rolling bearing fault identification. J. Sound Vib..

